# Hyperspectral estimation of chlorophyll content in jujube leaves: integration of derivative processing techniques and dimensionality reduction algorithms

**DOI:** 10.3389/fpls.2023.1260772

**Published:** 2023-11-14

**Authors:** Nigela Tuerxun, Jianghua Zheng, Renjun Wang, Lei Wang, Liang Liu

**Affiliations:** ^1^College of Geography and Remote Sensing Sciences, Xinjiang University, Urumqi, China; ^2^Xinjiang Key Laboratory of Oasis Ecology, Xinjiang University, Urumqi, China; ^3^Institute of Modern Forestry, Xinjiang Academy of Forestry Sciences, Urumqi, China

**Keywords:** hyperspectral data, elastic net, LASSO, support vector regression, invertible forest reflectance model, derivative processing

## Abstract

The leaf chlorophyll content (LCC) of vegetation is closely related to photosynthetic efficiency and biological activity. Jujube (*Ziziphus jujuba* Mill.) is a traditional economic forest tree species. Non-destructive monitoring of LCC of jujube is of great significance for guiding agroforestry production and promoting ecological environment protection in arid and semi-arid lands. Hyperspectral data is an important data source for LCC detection. However, hyperspectral data consists of a multitude of bands and contains extensive information. As a result, certain bands may exhibit high correlation, leading to redundant spectral information. This redundancy can distort LCC prediction results and reduce accuracy. Therefore, it is crucial to select appropriate preprocessing methods and employ effective data mining techniques when analyzing hyperspectral data. This study aims to evaluate the performance of hyperspectral data for estimating LCC of jujube trees by integrating different derivative processing techniques with different dimensionality reduction algorithms. Hyperspectral reflectance data were obtained through simulations using an invertible forest reflectance model (INFORM) and measurements from jujube tree canopies. The least absolute shrinkage and selection operator (LASSO) and elastic net (EN) were employed to identify the important bands in the original spectra (OS), first derivative spectra (FD), and second derivative spectra (SD). Support vector regression (SVR) was used to establish the estimation model. The results show that compared with full-spectrum modeling, LASSO and EN algorithms are effective methods for preventing overfitting in LCC machine learning estimation models for different spectral derivatives. The LASSO/EN-based estimation models constructed using FD and SD exhibited superior R^2^ compared to the OS. The important band of SD can best reveal the relevant information of jujube LCC, and SD-EN-SVR is the most ideal model in both the simulated dataset (R^2 ^= 0.99, RMSE=0.61) and measured dataset (R^2 ^= 0.89, RMSE=0.91). Our results provided a reference for rapid and non-destructive estimation of the LCC of agroforestry vegetation using canopy hyperspectral data.

## 1 Introduction

The leaf chlorophyll content (LCC) of vegetation is closely related to the absorption of carbon dioxide in the atmosphere and the process of photosynthesis, which is an indicator of the photosynthetic efficiency and biological activity of vegetation (Darvishzadeh et al., 2008). Conducting quantitative and real-time monitoring of chlorophyll content variations in forest could provide crucial information to understand the responses of ecosystems to changes in environmental, meteorological, and ecological factors (Zhen et al., 2021).

Jujube (*Ziziphus jujuba* Mill.) is a traditional economic forest tree species. Its fruit is sweet and juicy and is suitable for fresh food and dry processing. Jujube trees are known for their strong resistance to drought and wind, as well as their high yield and stability (Liu et al., 2020). As a result, they are becoming increasingly important in arid and semi-arid lands (Liu et al., 2020). Xinjiang produces half of the jujubes in China, and they are of higher quality compared to those planted in other regions (Bai et al., 2019). Non-destructive monitoring of LCC of jujube is of great significance for guiding agroforestry production and promoting ecological environment protection in arid and semi-arid lands.

Traditional laboratory LCC determinations are destructive and time consuming (Li et al., 2020). The development of remote sensing technology enables the acquisition of physical and chemical information of vegetation in a non-contact manner. Hyperspectral remote sensing technology is very effective for monitoring LCC due to its rapid and non-destructive capabilities (Shi et al., 2022). Compared with multispectral sensors, hyperspectral data, with its narrower bandwidth, provides distinct advantages for monitoring vegetation health by capturing different physical and chemical reactions in vegetation at various wavelengths (Jingguo et al., 2015; Ali and Imran, 2020). However, hyperspectral data contain much information and many bands; hence, some bands are highly correlated, which increases the redundancy of spectral information, leading to a distortion in prediction results and a reduction in prediction accuracy (Cheng et al., 2022). To address these issues, Sun et al. (2021) selected the first order derivative (FD) spectral data and using the correlation coefficient method to predict the LCC of maize. Lu and Peng (2015) calculated the correlation coefficient between vegetation index and chlorophyll concentration, finding that D715/D705 (D: first derivative), EBFR (simple ratios of the amplitude between the red and blue regions), D705/D722, and BND (normalized difference derivative at 722 and 700 nm) had a better estimation effect on chlorophyll concentration at the cherry leaf scale. Although the above methods have achieved better results, some limitations still exist. For instance, the method of setting the threshold based on the correlation between the band and chlorophyll content may ignore the collinearity of adjacent spectral data (Sun et al., 2021). The vegetation index method utilizes only one to four bands of information, which fails to fully capture the important information present in hyperspectral data (Lu and Peng, 2015). Studies have shown that the selection of important bands for modeling through dimensionality reduction algorithms typically yields equal or superior model prediction performance compared to full-spectrum models (Wang et al., 2022a; Zhu et al., 2022). The least absolute shrinkage and selection operator (LASSO) (Tibshirani, 1996) and elastic net (EN) (Zou and Hastie, 2005) are regularization methods that effectively reduce high-dimensional data by adjusting model parameters. These algorithms have demonstrated successful outcomes in various applications, including crop yield estimation (Cao et al., 2021b), leaf nitrogen estimation (Cao et al., 2021a) and forest biomass estimation (Takayama and Iwasaki, 2016). However, the potential of these two algorithms in estimating the LCC of agroforestry vegetation using hyperspectral data, such as jujube trees, remains unclear. FD and/or the second derivatives (SD) are commonly used spectral data preprocessing techniques (Wang et al., 2018b; Wang et al., 2021; Wang et al., 2022b). They are widely employed to mitigate noise, baseline effects, overlap problems, enhance spectral features, capture subtle details of spectral curves, and improve the accuracy of land surface parameter extractions (Li et al., 1993; Cui et al., 2022; Jin and Wang, 2022). However, to the best of our knowledge, there has been no research combining these two derivative processing techniques with LASSO and EN dimensionality reduction algorithms for predicting hyperspectral vegetation LCC.

Support vector regression (SVR), introduced by Cortes and Vapnik (Cortes and Vapnik, 1995) in 1995, is a versatile machine learning regression model. It has proven to enhance the efficiency of modeling vegetation physiological parameters while demonstrating improved stability in parameter estimation compared to other methods (Navarro et al., 2019; Liu et al., 2022). Therefore, this study aims to achieve the following objectives: (1) Propose a method for estimating LCC of agroforestry vegetation by integrating derivative processing techniques and dimensionality reduction algorithms, specifically utilizing FD and SD derivative processing along with LASSO and EN algorithms. (2) Compare the prediction performance of LCC using important spectral bands of different derivative orders (original spectra (OS), FD, SD) selected by LASSO and EN algorithms with the prediction performance of LCC based on modeling using full-spectrum data by establish SVR. (3) Evaluate the effectiveness of the proposed method using measured data and a substantial dataset of canopy reflectance data generated by the Invertible Forest Reflectance Model (INFORM), which accurately represents the annual growth stages of jujube trees. This analysis will help assess the practicality and applicability of the proposed approach.

## 2 Materials and methods

### 2.1 Study area

Ruoqiang County is located in southeastern Xinjiang, the southeastern margin of the Taklimakan Desert, and the eastern Tarim Basin. It is located between 86°45’-93°45’ E and 36°05’-41°23’ N, with an altitude of 846-4500 m and a total area of 202,300 square kilometers. Ruoqiang County is the largest county in China. It has a warm temperate continental desert arid climate, providing unique natural conditions for the development of the agroforestry vegetation (Cui, 2019). Among them, the ‘Huizao’ variety of *Z. jujuba* Mill. is a well-known product in Xinjiang.

**Figure 1:**
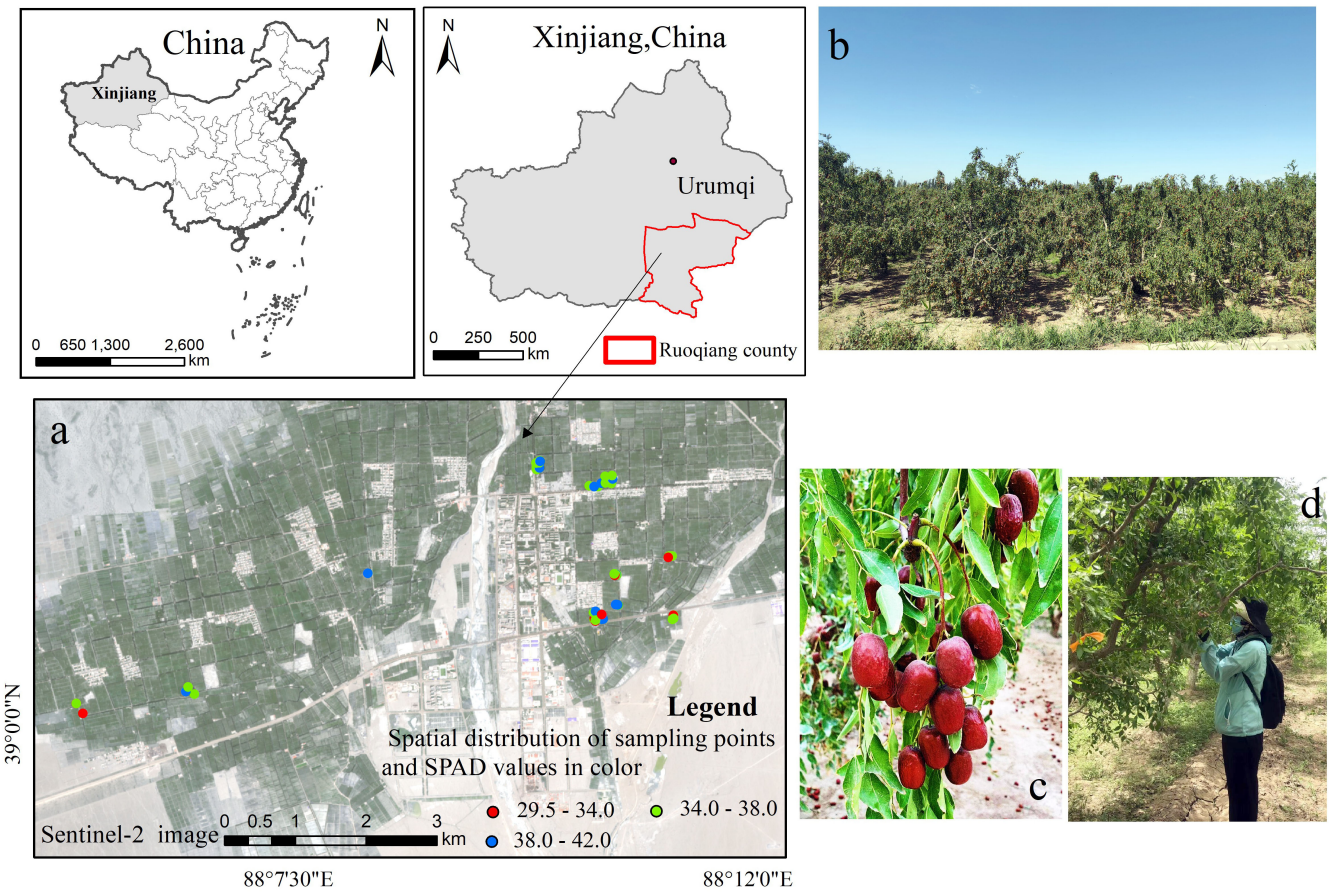
Study area locations: **(A)** sampling plots (SPAD value refers to leaf relative chlorophyll content); **(B)** jujube orchard; **(C)** mature state of the ‘Huizao’ variety of *Z. jujuba* Mill; **(D)** research team collecting jujube leaves.

A total of 69 samples were collected in the study area. The minimum interval of sample points is more than 15m.The location of study area is shown in **Figure 1**.

### 2.2 Data collection and preprocessing

#### 2.2.1 Measurement of hyperspectral reflectance

A portable spectroradiometer, the PSR-3500 manufactured by Spectral Evolution, USA, was used to measure the canopy spectra of 69 sample points (trees). The spectral reflectance data were obtained from May 19th to June 1st, 2020, during the flowering stage of jujube trees, between 11:00 and 17:00 Beijing time, under clear, windless, and cloudless conditions (Cui et al., 2022). The spectral range covered 350-2500 nm with a 1 nm interval, resulting in 2150 wavebands. The spectrometer probe was vertically downward during each measurement. This measurement process was repeated 5 times, and the average of the spectral curve from these repetitions was taken as the reflectance of the sample. A total of 345 spectral data were measured. To account for any variations in the ambient radiation, we employed the white reference panel to calibrate the instrument prior to each reflectance measurement. To reduce noise interference caused by atmospheric scattering and other reasons (Badola et al., 2022), only the 350-1050 nm band range is retained, and savitzky-golay smoothing with a second-order polynomial and window size of 5 (Cai et al., 2019) was applied to smooth and denoise the spectral data.

#### 2.2.2 Measurement of leaf relative chlorophyll content

**Figure 2:**
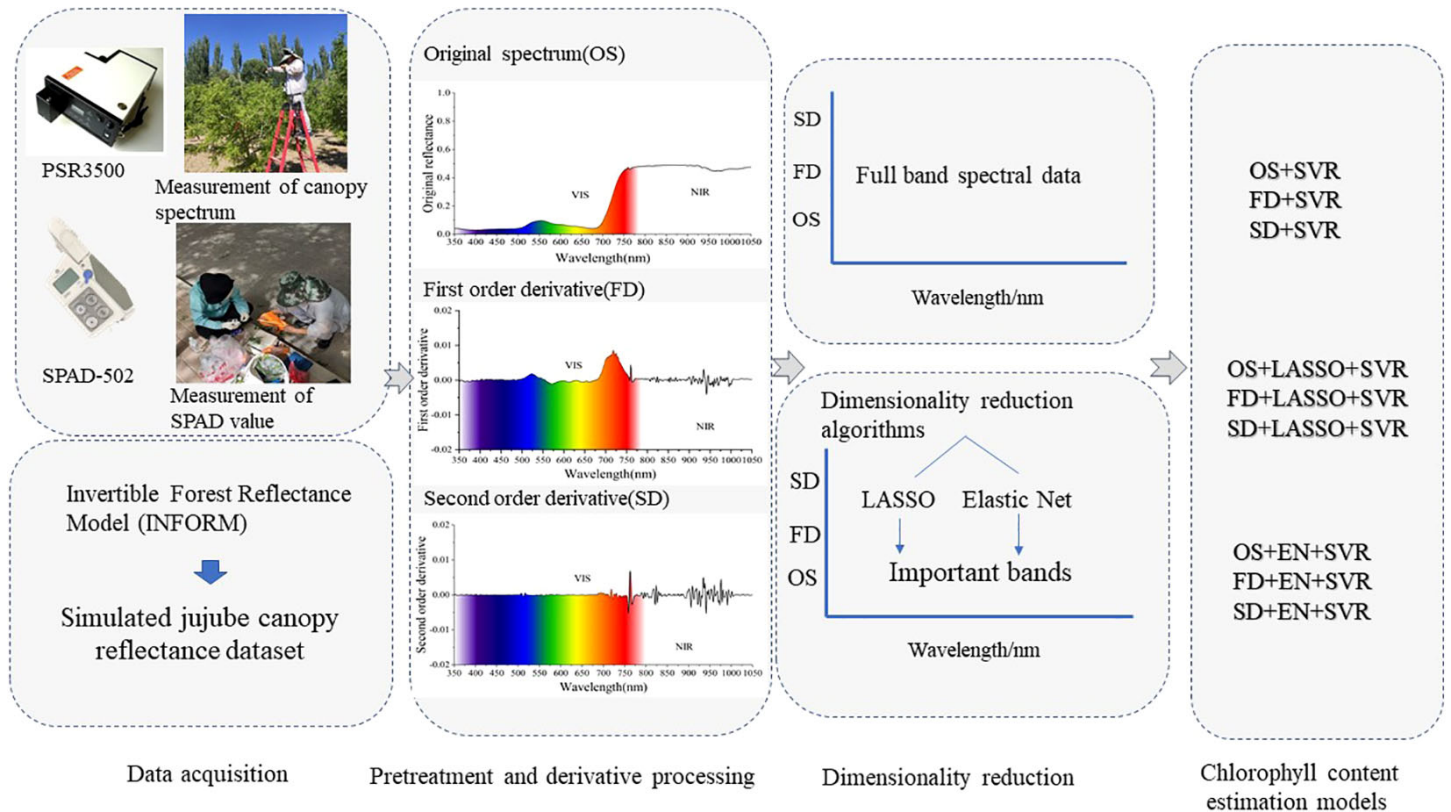
The diagram of the study.

The soil–plant analyzer development (SPAD)-502 Plus portable chlorophyll meter(Konica Minolta, Japan) measures the transmittance in the red region (650 nm) and infrared region (940 nm) through the leaf, providing a correspondence value of chlorophyll content in three significant digit values (leaf relative chlorophyll content), thereby characterizing the chlorophyll content in leaves (Yang et al., 2021). The measurement of SPAD values was carried out concurrently with canopy spectral measurements, two leaves were taken from each jujube tree in the east, west, south, and north, as well as vertical ground directions, resulting in a total of 10 leaves per tree. During the measurements, the leaf veins were avoided, and the SPAD value was recorded five times at different positions along the leaf, from the base to the tip. The average of the SPAD measurements for the 10 leaves was considered as the SPAD value for the sampled tree. The measurement processes of canopy spectral and SPAD value are illustrated in the data acquisition section of **Figure 2**.

Existing research (Zhang et al., 2022) has established and validated the formula (LCC(µg/cm^2^))= 0.709 * SPAD – 1.576) for a robust conversion (R^2 ^= 0.52) of SPAD values to LCC. In this paper, SPAD values are utilized to characterize the LCC in the measurement dataset.

#### 2.2.3 Simulation of hyperspectral reflectance in jujube tree canopies

A simulated hyperspectral dataset of the jujube canopy was generated using the Invertible Forest Reflectance Model (INFORM) (Atzberger, 2000), which combines the Forest Light Interaction Model (FLIM), Scattering by Arbitrary Inclined Leaves (SAILH), and PROSPECT model. The dataset was simulated using a range of input parameters listed in **Table 1**. Based on previous studies (Lu et al., 2022; Wu et al., 2023) on SPAD estimation of jujube trees at different growth stages and the existing conversion relationship between LCC and SPAD values, the Cab value was set to be 25-50 ug/cm^2^ to represent the total growth stage of jujube. N represents the leaf structure parameter, and N is the number of compact layers specifying the average number of air/cell walls interfaces within the mesophyll (Jacquemoud et al., 2009). Usually, the N values of trees fall within the range of 0.63-3, and most studies use a fixed N value (Hernandez-Clemente et al., 2014; Yuan et al., 2015; Brown et al., 2019; Shi et al., 2022). As trees age, N values tend to increase. To ensure diversity in leaf sample types and statuses, this study has set the range for N values between 1 and 1.5. Scale factor for soil reflectance is the spectral reflectance of the underlying (uncovered) soil (Atzberger, 2000). Based on the empirical knowledge from our research team’s field investigations, we have observed that the soil conditions in jujube orchards are quite complex. Typically, the soil in jujube orchards is not entirely covered by trees and other vegetation. On poorly managed land, there may even be situations where the soil is completely exposed. Drawing from previous studies (Hernandez-Clemente et al., 2014; Darvishzadeh et al., 2019; Zarco-Tejada et al., 2019) on other tree species, we have decided to set the scale factor for soil reflectance range between 0.6 and 1. This range represents variations in soil parameters during the growth of jujube trees. This parameter helps us more accurately simulate and describe the soil conditions in jujube orchards, enhancing the effectiveness of our simulation data. Other parameters (Single trees LAI (m^2^·m^−2^) and Average leaf angle of tree canopy) were set within a reasonable range according to the field investigation of the research team. Ultimately, a total of 2100 spectra were generated, covering all possible combinations of the input parameters.

**Table 1:** INFORM model parameters that were used in this study to simulate the canopy reflectance spectra.

Parameter	Minimum	Maximum	Step
Cab(ug/cm^2^)	25	50	5
The leaf structure parameter(N)	1	1.5	0.5
Single trees LAI (m^2^·m^−2^)	1	7	1
Scale factor for soil reflectance	0.6	1	0.1
Average leaf angle of tree canopy(degree)	0	60	15

(Other ten fixed parameters are: solar zenith angle = 30°; Observer zenith Angle = 0°; azimuth angle = 0°; fraction of diffuse incident radiation is 0.1; Equivalent water thickness is 0.03 g·cm^−2^; Dry matter content is 0.012 g.cm-2; Stem density is 500 ha^−1^; Tree height is 2.5m; Crown diameter is 2m; LAI understory is 0.1 m^2^·m^−2^).

### 2.3 Modeling process

In this study, estimation models were established by integrating OS, FD, and SD spectra, dimensionality reduction algorithms (LASSO, EN) and SVR. The modeling process was as follows: first, the SVR was used to model the OS, FD, and SD spectra for the full-spectrum prediction of LCC. Second, the LASSO and EN were employed to reduce the dimensions of the OS, FD and SD spectral data and SVR was performed on the important bands after dimension reduction. A total of nine estimation models were established. The hyperspectral simulated dataset obtained from the radiative transfer model INFORM and the measured dataset were used to test the validity of the proposed integration method. Modeling process of this research is shown in **Figure 2**.

### 2.4 Derivative processing

The utilization of derivative techniques has become prevalent in mitigating noise, baseline effects, overlap problems, enhancing spectral features, capturing subtle details of spectral curves, and improving the accuracy of land surface parameter extractions(Wang et al., 2018b). Not only is the derivative technique a potent tool for spectral analysis, it can also tackle collinearity concerns (Wang et al., 2021). In the present study, FD and SD were employed to process the hyperspectral data with the aid of Origin 2021 (OriginLab).

### 2.5 Dimensionality reduction algorithms

#### 2.5.1 LASSO

LASSO, proposed by Robert Tibshirani in 1996 (Tibshirani, 1996), is a biased estimation method for compressing model coefficients and variable selection. LASSO adds the L1 norm penalty term on the basis of the least squares method to compress the estimated parameters. When the sum of the absolute values of the regression coefficients is less than a constant, the sum of squared residuals is minimized to obtain regression coefficients equal to 0; thus, the effects of independent variables with little or no influence are compressed to zero. The multiple linear model can be expressed as follows:


(1)  y=Xβ+ϵ

where β is the linear variable, X is the independent variable (that is, the hyperspectral data), y is the dependent variable (that is, the jujube SPAD value), and ϵ is the error.

From Equation (1), the estimation of parameter β can be expressed as follows:


(2) $$J\left(\beta \right)=\sum^{}{\left(y-X\beta \right)}^{2}$$

Compared to linear regression, in LASSO, an L1 penalty term is added as follows:


(3) $$J\left(\beta \right)=\sum^{}{\left(y-X\beta \right)}^{2}+\lambda ∥\beta {∥}_{1}=\sum^{}{\left(y-X\beta \right)}^{2}+\sum^{}\lambda \left|\beta \right|$$

where λ  is a regularization parameter and λ≥0 . The penalty strength of the model is related to the regulation parameter λ . Variable screening can be achieved by controlling the adjustment parameter λ

#### 2.5.2 Elastic net

Zou and Hastie proposed the EN technique (Zou and Hastie, 2005), which integrates the characteristics of ridge and LASSO, and the penalty term has both an L1 norm term and an L2 norm term. The EN includes a mixture parameter α, which is selected based on the criterion of minimizing the MSE of the training samples and the MSE of the prediction bias. α is a number between 0 and 1 that acts in conjunction with λ to regulate the size of the penalty term. The estimation of parameter β can be expressed as follows:


(4) $$\begin{array}{c} J\left(\beta \right)=\sum^{\;}{\left(y-X\beta \right)}^{2}+\alpha \lambda ∥\beta {∥}_{1}+\frac{1-\alpha }{2}\lambda ∥\beta {∥}_{2}^{2}\\ =\sum^{\;}{\left(y-X\beta \right)}^{2}+\lambda \sum^{\;}\left(\alpha \left|\beta \right|+\left(1-\alpha \right)\beta^{2}\right) \end{array}$$

It has been proven that the LASSO and EN models are more interpretable when using the value of λ with the minimum MSE than when using the value of λ with the minimum SE (Cao et al., 2021a). Therefore, the value of λ with the minimum MSE is chosen in this paper.

### 2.6 SVR

The support vector machine (SVM) theory proposed by Vapnik was initially used for supervised classification processes(Cortes and Vapnik, 1995). SVR is the regression method of SVM, the idea of SVR has been described by Smola and Schölkopf (Smola and Schölkopf, 2004). In SVR, the mapping of input data in higher-order feature space is accomplished by several types of kernel functions (Li et al., 2021), such as linear, nonlinear, sigmoid, polynomial, and radial basis functions (RBFs). Among various kernel functions, the RBF kernel can achieve good results. Therefore, we adopted the RBF kernel of SVR, where the hyperparameters (C and gamma) were used for cross-validated grid search, parameter tuning and model training in R software.

### 2.7 Model evaluation method

The determination coefficient (R^2^), root mean square error (RMSE) are compared to evaluate and optimize the model accuracy. The higher the R^2^ value is, the stronger the prediction ability of the model. The smaller the calculated values of RMSE, the higher the prediction accuracy of the model. The calculation formula is as follows:


(5) $$R^{2}={\left[\frac{\sum_{i=1}^{n}(y_{i}-\bar{y})(f_{i}-\bar{f})}{\sqrt{\sum_{i=1}^{n}{\left(y_{i}-\bar{y}\right)}^{2}{\left(f_{i}-\bar{f}\right)}^{2}}}\right]}^{2}$$

(6) $$RMSE=\sqrt{\frac{1}{n}\sum_{i=1}^{n}{\left(y_{i}-f_{i}\right)}^{2}}$$

where n is the number of samples, $y_{i}\left(i=1,\ldots .,n\right)$ is the measured value, $f_{i}\left(i=1,\ldots .,n\right)$ represents the predicted value, $\;\bar{y}$ represents the average observed value, and $\bar{f}$ represents the average predicted value.

## 3 Results

### 3.1 Statistical description of measured and simulated dataset

The statistical analysis of measured dataset and simulated dataset are presented in **Table 2**. A total of 69 samples were collected in measured dataset; the mean SPAD value was 36.00, the range was 29.50 to 42, the median was 35.70, the interquartile range (Q3-Q1) was 3.1, the standard deviation was 2.69, and the coefficient of variation was 0.07. The 69 samples were randomly split into a training set and a validation set at a ratio of 75%:25%. The training set consisted of 52 samples, while the validation set comprised 17 samples.

**Table 2:** Statistical analysis of measured dataset and simulated dataset.

Dataset	N	Min	Q1	Median	Q3	Max	Mean	SD	CV
Measured dataset (SPAD)	69	29.50	34.60	35.70	37.70	42.00	36.00	2.69	0.07
Measured training dataset	52	29.50	34.10	35.30	37.30	42.00	35.60	2.80	0.08
Measured validation dataset	17	34.70	35.6	37.00	38.40	41.20	37.30	1.89	0.05
Simulated dataset (LCC)	2100	25.00	30.00	37.50	45.00	50.00	37.50	8.54	22.8
Simulated training dataset	1569	25.00	30.00	40.00	45.00	50.00	37.50	8.52	22.7
Simulated validation dataset	531	25	30.00	35.00	45.00	50.00	37.49	8.62	23.0

N represents the size of the dataset; SD indicates the standard deviation; CV stands for the coefficient of variation.

A total of 2100 samples were collected in simulated dataset; the mean LCC was 37.5 ug/cm^2^, the range was 25 ug/cm^2^to 50 ug/cm^2^, the median was 37.5 ug/cm^2^, the interquartile range (Q3-Q1) was 15 ug/cm^2^, the standard deviation was 8.54 ug/cm^2^, and the coefficient of variation was 22.5 ug/cm^2^. The coefficient of variation was calculated to be 0.07. The simulated dataset of 2100 hyperspectral data were randomly divided into validation set and training set in the same proportion.

### 3.2 Results of spectral dimensionality reduction

**Figure 3:**
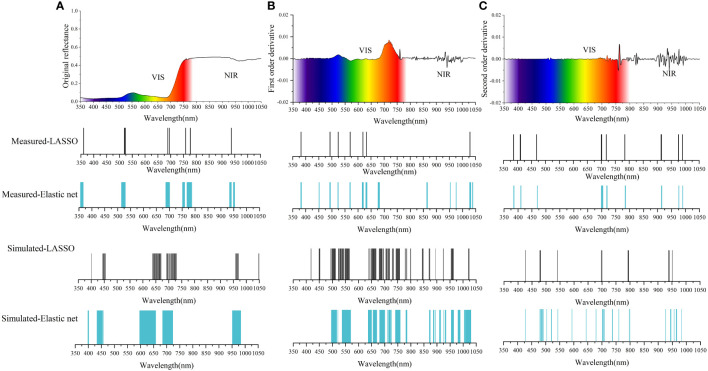
Distribution of important bands screened by the LASSO and EN: **(A)** selection based on OS; **(B)** selection based on FD; **(C)** selection based on SD.

**Figures 3A-C** show the important band distribution of the OS, FD and SD spectra after dimension reduction by LASSO and EN. For the measurement data: There were 10 important bands selected in OS-LASSO, 7 important bands were selected in FD-LASSO, and 13 important bands were selected in SD-LASSO. The optimization function *J(β)* of the EN contains coefficients α (0<α<1). In this paper, the range of α (0-1) is divided into 100 parts: the larger α is, the fewer variables are selected, and the smaller α is, the more variables are selected. The α values calculated for the OS, FD and SD spectra were 0.21, 0.94 and 0.52, respectively. Thus, 80, 11, and 30 bands were selected when using EN. The results of the simulated dataset show that 97 important bands are selected by the OS-LASSO method, 161 important bands are selected by FD-LASSO, and 31 important bands are selected by SD-LASSO. The α values calculated for the OS, FD and SD spectra were 0.50, 0.20 and 0.91, respectively. Thus, 172, 208, and 40 bands were selected when using EN.

### 3.3 Model building and evaluation

**Figure 4:**
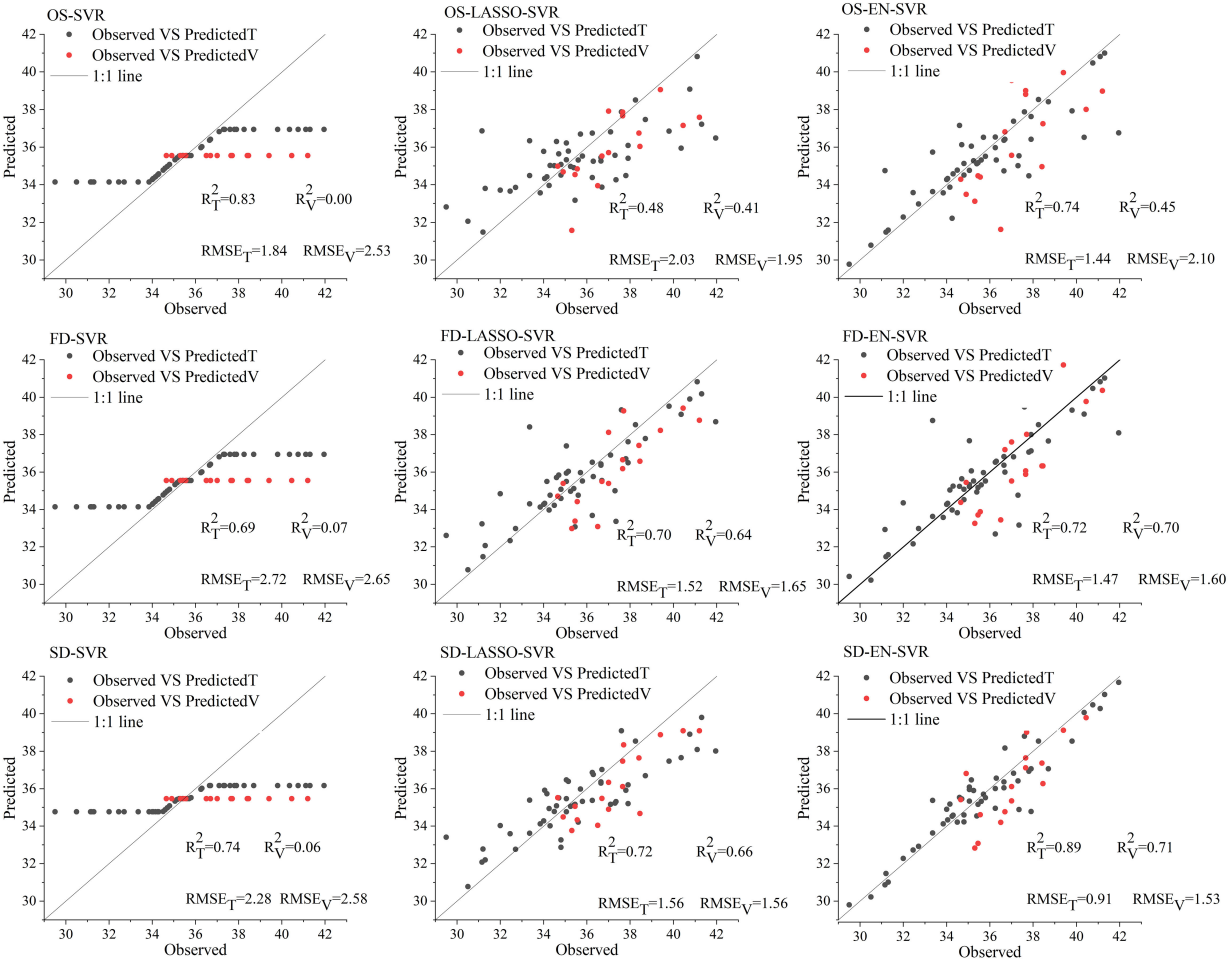
Scatterplots of the measured SPAD values vs. predicted SPAD values using SVR (T=training set, V=validation set).

**Table 3** outlines the detailed outcomes of measured dataset and simulated dataset generated by the SVR. The nine SVR results indicated that the SVR model based on SD-EN-SVR achieved the best quantitative prediction accuracy in both the measured (R^2 ^= 0.89, RMSE=0.91) and simulated datasets (R^2 ^= 0.99, RMSE=0.61). In addition, the model fitting effect based on SD and FD was better than the model fitting effect based on OS, indicating that the FD and SD processing is more effective than directly modeling with the OS. The scatter plot (**Figure 4**) of the measured versus predicted SPAD values demonstrates that SD-EN-SVR achieved the best estimation results with a fitting line close to the 1:1 line; therefore, this model has strong stability and high predictive ability. In contrast, the validation SPAD estimation deviated from the 1:1 line in the SVR model based on full spectra of OS, FD, and SD. It can be concluded that the SVR model was not suitable for processing high-dimensional data, and the result was overfitting. The combination of LASSO and EN with SVR significantly improved the overfitting phenomenon, and the R^2^ of the validation set was no longer less than 0.1.

**Table 3:** The evaluation of nine models (T=training set, V=validation set).

Model	Measured dataset				Simulated dataset			
	R^2^_T_	R^2^_V_	RMSE_T_	RMSE_v_	R^2^_T_	R^2^_V_	RMSE_T_	RMSE_v_
OS-SVR	0.83	0.00	1.84	2.53	0.77	0.02	8.67	8.76
FD-SVR	0.69	0.07	2.72	2.65	0.77	0.06	8.67	8.77
SD-SVR	0.74	0.06	2.28	2.58	0.98	0.01	2.39	8.61
OS-LASSO-SVR	0.48	0.41	2.03	1.95	0.79	0.78	4.94	5.14
FD-LASSO-SVR	0.70	0.64	1.52	1.65	0.94	0.93	2.22	2.47
SD-LASSO-SVR	0.72	0.66	1.56	1.56	0.95	0.94	1.90	2.20
OS-EN-SVR	0.74	0.45	1.44	2.10	0.88	0.87	3.26	3.47
FD-EN-SVR	0.72	0.70	1.47	1.60	0.97	0.97	1.43	1.60
SD-EN-SVR	0.89	0.71	0.91	1.53	0.99	0.97	0.61	1.07

## 4 Discussion

### 4.1 Effect of important bands on SPAD values estimation

Hyperspectral data provide ground object reflection information in thousands of bands. Directly using machine learning method such as SVR to process the full-spectrum information may lead to overfitting of the model (**Table 2**; **Figure 4**). Improving the accuracy of prediction models while maintaining effective spectral information is a challenging problem. In this paper, LASSO, and EN were adopted to reduce the complexity of the model and prevent overfitting. The LASSO compresses the bands to a greater extent compared to EN, and the EN is more moderate than the LASSO, and the selected bands are more uniform (**Figure 3**). It is worth noting that the model established by the bands selected by the EN is more stable and accurate than LASSO in LCC estimation. This may be because the EN combines the characteristics of ridge regression and LASSO, and the penalty term has both L1-norm and L2-norm terms. Furthermore, important bands selected based on the EN method were concentrated in the red-edge region (670-760 nm), defined as the boundary between chlorophyll absorption in the red and the onset of leaf scattering in the near-infrared light (Curran et al., 1990). Studies have proved that the red-edge bands are closely related with vegetation LCC and is more sensitive for detecting slight changes in LCC than that in the green region (500-560 nm) of the spectrum (Ju et al., 2010; Delegido et al., 2011; Li et al., 2016).

**Figure 3** illustrates that the important bands obtained through LASSO and EN from the measurement dataset closely resemble those from the simulation dataset. Bands around 700 nm are selected in both datasets in the original spectrum. In the First Derivative (FD) spectrum, both datasets primarily select bands in the 500-550 nm and 600-700 nm ranges. In the FD spectrum, both datasets select most of the bands in the 500-550 nm and 600-700 nm ranges. In the SD spectrum, the spectra obtained from the measurement dataset and the simulation dataset are mainly distributed at 425 nm, 700 nm, and 900-975 nm. The method proposed in this study is relatively stable in the important bands selection of different datasets and the results of these important bands can provide a reference for wavelength selection in developing LCC detection equipment in the future.

### 4.2 Effect of derivative treatment

In previous studies, various methods, such as enhancement transform, curve smoothing, continuous curve removal, wavelet-based noise removal, have been commonly used to optimize hyperspectral data to improve the estimation accuracy of vegetation parameters (Wang et al., 2018a). Among them, derivative transformation was used as a robust mathematical analysis tool for processing data such as hyperspectral and remote sensing images because of its advantages of reducing noise and enhancing the details of data (Jin and Wang, 2016; Qu and Liu, 2017). This study proved that the combination of derivative spectral processing and dimensionality reduction algorithms effectively improved the estimation performance of LCC compared with the original spectral data modeling. In this study, FD and SD spectra were more robust than OS during modeling. Compared with OS modeling using the same combination of “EN/LASSO-SVR”, the modeling accuracy after derivative processing is greatly improved (**Figure 4**). However, the FD spectra did not perform well compared to the SD spectra. This pattern occurs because the SD spectra enhance the peaks and valleys in the OS, distinguishing them from noise and enabling more accurate isolation and quantification of the vegetation signal than with the FD (Xiao-chen et al., 2008; Zhang et al., 2018). Additionally, the SD removes more of the soil background effects than the FD (Thorp et al., 2004), which can further improve the accuracy of LCC value estimation.

In Section 3.3, we presented the results of model building and evaluation, where the combination of LASSO and EN with SVR demonstrated relatively favorable performance. However, one notable issue that emerged was the consistent underestimation of accuracy on the validation set. From the model fitting results of the measured dataset presented in **Figure 4**, it can be observed that the LASSO/EN+SVR models built on FD and SD spectra exhibit a relatively better performance in mitigating underestimation of validation set compared to those built on OS spectra. This phenomenon may be attributed to the lower data quality of the OS spectral dataset, whereas FD and SD spectra are more reliable. This highlights the significance of employing FD and SD spectral preprocessing when utilizing LASSO/EN+SVR models. It can be seen from **Table 3** that the problem of low accuracy in the validation set is greatly improved on the simulated data set. For instance, the difference in R^2^_T_ and R^2^_V_ between OS-LASSO-SVR, FD-LASSO-SVR, SD-LASSO-SVR, and OS-EN-SVR is only 0.1, while FD-EN-SVR achieves R^2^_T_ and R^2^_V_ values of 0.97. These findings suggest that the canopy information from the measured data is more intricate, potentially contain more information on soil and environmental factors. Consequently, the accuracy of the validation set may need to be moderately sacrificed to counteract the influence of complex environmental factors. This phenomenon emphasizes the importance of FD and SD derivative preprocessing in future research based on measured data.

### 4.3 Model uncertainty analysis

This paper presents an integration of derivative processing and dimensionality reduction algorithms method for estimating chlorophyll content in jujube leaves based on hyperspectral data and achieves good results in both measured and simulated datasets. The measured dataset in this study was collected during the flowering stage of jujube trees, during which the canopy reflectance of jujube trees was greatly affected by soil background and canopy structure (Yu et al., 2014). Despite these influences, the proposed combination method of derivative processing and dimensionality reduction algorithms in this paper still achieved favorable results during the flowering stage of jujube trees.

The chlorophyll content, leaf area index and other parameters (scale factor for soil reflectance and average leaf angle of tree canopy) of the simulated data have a wide range, which could represent the growth state of jujube during the whole growth stage. Therefore, the simulated dataset proves the validity of the integration method proposed in the study and generalizes the obtained results. This paper has contributed to the establishment of a prediction model of chlorophyll content in jujube leaves, but there are still the following limitations: (1) This study was based on hyperspectral data collected on the ground and simulated using the INFORM model, not combined with image data. Therefore, the effect of the results on the UAV and satellite scale needs to be verified. (2) The derivative processing method used in this study can be further optimized. The fractional derivative spectral data processing method has achieved good results in hyperspectral estimation of soil salinization (Wang et al., 2018b) and soil total nitrogen content (Yang et al., 2022), However, the effect of improving the estimation accuracy of chlorophyll content in combination with dimensionality reduction algorithms needs to be further explored in the future. (3) In this study, we did not conduct year-round destructive experiments to directly establish the conversion relationship between LCC and SPAD values. However, future research efforts will focus on conducting such experiments at various growth stages to improve the precision of LCC estimation. (4) In future research, we plan to collect data throughout the entire growth stage of jujube trees, taking into account variations in soil background and canopy structure at different stages.

## 5 Conclusion

In this study, we combined the derivative processing techniques and dimensionality reduction algorithms to improve the hyperspectral estimation of jujube LCC. The main results were as follows: (1) LASSO and EN algorithms are effective methods for preventing overfitting in LCC machine learning estimation models for different spectral derivatives. (2) The LASSO/EN-based estimation models constructed using FD and SD exhibited superior R^2^ compared to the OS. (3) The important bands of the SD can effectively reveal the relevant information of LCC. In both the dataset simulated by the radiative transfer model INFORM, which represents the canopy reflectance of jujube trees throughout the entire growth stage (R^2 ^= 0.99, RMSE=0.61), and the measured dataset collected during the flowering stage of jujube with the interference from soil background and canopy structure (R^2 ^= 0.89, RMSE=0.91), the SD-EN-SVR model demonstrates the highest performance and is considered the most optimal model. This study provides a convenient method to estimate agroforestry vegetation parameters from canopy hyperspectral data and can provide a scale conversion reference for the LCC estimation of UAV and satellite remote sensing.

## Data availability statement

The original contributions presented in the study are included in the article/supplementary material, further inquiries can be directed to the corresponding author/s.

## Author contributions

NT: Writing – original draft, Writing – review & editing. JZ: Conceptualization, Supervision, Writing – review & editing. RW: Formal Analysis, Investigation, Software, Writing – review & editing. LW: Software, Writing – review & editing. LL: Methodology, Software, Writing – review & editing.
